# Knowledge, Attitudes and Practices on the Use of Antidiabetic Medications for Weight Loss: A Cross‐Sectional Study in the Lebanese Population

**DOI:** 10.1002/edm2.70286

**Published:** 2026-07-14

**Authors:** Rim Masri, Hekmat Kaakour, Hasnaa Hamdan, Pascale Salameh

**Affiliations:** ^1^ Faculty of Medical Sciences Lebanese University Hadath Lebanon; ^2^ Department of Endocrinology, Faculty of Medical Sciences Lebanese University Hadath Lebanon; ^3^ MEDICA Research Investigation Hadath Lebanon; ^4^ INSPECT‐LB (Institut National de Santé Publique, d'Épidémiologie Clinique et de Toxicologie‐Liban) Beirut Lebanon

**Keywords:** antidiabetic, attitudes, knowledge, Lebanon, obesity, practices, weight loss

## Abstract

**Background:**

The growing use of antidiabetic medications for weight loss has raised concerns about public awareness, safety and appropriate use. This study assessed knowledge, attitudes and practices (KAP) regarding antidiabetic medications use for weight loss among Lebanese adults and identified associated factors.

**Methods:**

A cross‐sectional online survey was conducted among Lebanese adults between June and July 2025 using convenience sampling through social media. The questionnaire was adapted from published instruments, expert‐reviewed and pilot‐tested. Data on sociodemographic characteristics, KAP and medication use were collected. Knowledge and attitude scores were calculated, and factors associated with medication use were evaluated by multivariable regression.

**Results:**

A total of 405 participants were included. Mean knowledge and attitude scores were 69.39 ± 8.71 and 69.11 ± 5.54 (out of 100), indicating moderate knowledge and cautious attitudes. Higher education and healthcare‐related employment were independently associated with higher knowledge and more appropriate attitudes toward safe use of antidiabetic medications, whereas previous weight‐control complications were associated only with more appropriate attitudes. Overall, 12.3% reported using antidiabetic medications for weight loss, primarily semaglutide. Among users, 62.0% experienced side effects, 46.0% reported weight regain after discontinuation, and 32.0% used these medications without medical consultation. Medication use was associated with smoking (aOR 3.13, 95% CI 1.53–6.38), prior weight‐control complications (aOR 8.50, 95% CI 3.27–22.12), higher knowledge scores (aOR 1.05, 95% CI 1.00–1.09), and adherence to a weight‐loss diet (aOR 2.21, 95% CI 1.35–3.60), while higher occupational physical activity was inversely associated (aOR 0.58, 95% CI 0.41–0.84).

**Conclusion:**

Lebanese adults demonstrated moderate knowledge and generally appropriate attitudes toward the safe use of antidiabetic medications for weight loss. Reported side effects, weight regain and unsupervised use underscore the need for public education, physician‐guided counselling and stronger oversight. Findings should be interpreted considering the cross‐sectional design and convenience sampling, limiting causal inference and generalizability.

## Graphical abstract

Knowledge of antidiabetic medications for weight loss was moderate among Lebanese adults, while attitudes were generally positive. Medication use was associated with higher body mass index, chronic disease and social influences, highlighting the need for targeted education and evidence‐based guidance to promote safe and informed use.

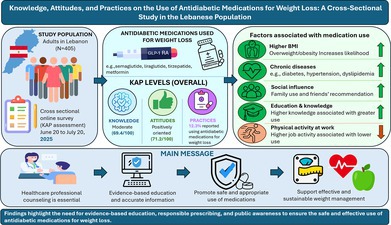

**Abbreviations**
BMI: body mass index
CI: confidence interval
FDA: Food and Drug Administration
GLP‐1 RAs: glucagon‐like peptide‐1 receptor agonists
IRB: Institutional Review Board
KAP: knowledge, attitudes and practices
OR: odds ratio
SD: standard deviation
WHO: World Health Organization

## 1 Introduction

Obesity is a major global public health challenge and is defined as a body mass index (BMI) ≥ 30 kg/m^2^, while overweight is classified as a BMI between 25 and 30 kg/m^2^. Obesity is associated with increased morbidity and mortality worldwide [1, 2]. Excess adiposity, particularly accumulation of visceral fat, is strongly linked to metabolic and cardiovascular complications, including type 2 diabetes mellitus, hypertension, dyslipidemia and cardiovascular disease [2, 3]. Consequently, maintaining a healthy body weight and limiting visceral fat accumulation are essential strategies for reducing obesity‐related health risks [2, 3].

In Lebanon, the burden of obesity has increased substantially over recent decades. According to the Global Nutrition Report, 39.9% of adult women and 30.5% of adult men are classified as obese, compared with regional Middle Eastern averages of 10.3% among women and 7.5% among men [4]. National epidemiological studies further confirm the high prevalence of obesity and its associated comorbidities among Lebanese adults [5]. This rising prevalence of obesity has been accompanied by a growing burden of metabolic disorders, with diabetes affecting up to 14.3% of women and 17.7% of men, thereby compounding public health challenges in the country [4].

Considering this situation, multiple weight management approaches have been adopted, including lifestyle modification, pharmacotherapy and bariatric surgery. Although lifestyle interventions remain the cornerstone of obesity management, pharmacological approaches have gained increasing popularity, particularly among individuals who struggle to achieve sustained weight loss through diet and physical activity alone [6, 7].

In recent years, antidiabetic medications, especially glucagon‐like peptide‐1 receptor agonists (GLP‐1 RAs) such as semaglutide (Ozempic), liraglutide (Saxenda) and tirzepatide (Mounjaro), as well as other antidiabetic agents such as metformin (Glucophage), have emerged as important pharmacological options for weight management. While primarily approved for glycemic control in individuals with type 2 diabetes, some of these agents are approved for weight loss at specific doses, whereas others are increasingly used off‐label for weight reduction among individuals without diabetes [6, 7]. Their mechanisms of action, including appetite suppression, delayed gastric emptying, and improvements in glycemic and metabolic regulation, have contributed to their widespread adoption [7].

Despite their demonstrated efficacy, concerns persist regarding the safety, appropriate indications and long‐term consequences of using antidiabetic medications for weight loss. Reported adverse effects include gastrointestinal symptoms, gallbladder disease, acute kidney injury, pancreatitis and thyroid C‐cell tumours observed in rodent studies, although a causal relationship has not been established in humans [7, 8]. Recent reports have additionally highlighted concerns regarding unsupervised use of GLP‐1 RAs for cosmetic or non‐medically indicated weight loss, emphasizing the importance of appropriate patient selection, counselling and follow‐up [9]. Moreover, increasing demand for GLP‐1 RAs has been associated with concerns regarding off‐label use, self‐medication practices and medication shortages affecting patients with approved indications, highlighting the need for stronger regulatory oversight and responsible prescribing practices [10].

The rapid rise in public interest surrounding GLP‐1 RAs has also coincided with the growing role of digital and social media platforms. Recent analyses of online discussions have shown that social media plays a significant role in shaping public perceptions of the effectiveness, safety and accessibility of these medications [11]. Similarly, large‐scale evaluations of social media content have demonstrated a marked increase in online engagement related to GLP‐1 RAs, reflecting growing public interest in both approved and off‐label uses for weight loss [12]. While these platforms may increase awareness, they may also contribute to the dissemination of incomplete or inaccurate information, potentially encouraging inappropriate medication use and unrealistic expectations regarding weight‐loss outcomes.

The Lebanese context may present unique challenges regarding the use of these medications. Ongoing economic instability, variations in healthcare access, widespread use of social media for health‐related information, and inconsistent regulatory oversight may be associated with public perceptions, attitudes and practices related to pharmacological weight management. Understanding how the Lebanese population perceives and uses antidiabetic medications for weight loss is therefore important for informing public health interventions, clinical counselling strategies and regulatory policies.

Several studies conducted in the Middle East and North Africa (MENA) region have explored knowledge, attitudes and practices (KAP) regarding weight management medications and GLP‐1 RAs [13, 14, 15, 16, 17]. Abutaima et al. [13] reported moderate knowledge levels and generally cautious attitudes toward GLP‐1 RAs among the Jordanian population. Similar findings were reported among Saudi populations by Algarni et al. and AlGamdi [14, 15]. However, these studies were conducted in different healthcare systems and sociocultural settings and may not be directly generalizable to Lebanon. Furthermore, other investigations, such as that of Li et al. [16], focused on patients with diabetes rather than the public. Available Lebanese research has primarily focused on healthcare professionals. For example, Hijazi et al. [17] found that community pharmacists generally possessed adequate knowledge regarding medications used for weight management but frequently dispensed them without prescription or comprehensive patient assessment.

Despite growing use of antidiabetic medications for weight loss, evidence regarding public KAP in Lebanon remains scarce. To our knowledge, no nationwide study has comprehensively evaluated KAP regarding the use of antidiabetic medications for weight loss among Lebanese adults. Furthermore, little is known about the extent of medication use, sources of information, safety perceptions and factors associated with their use in the Lebanese population. Therefore, this study aimed to assess knowledge, attitudes and practices regarding the use of antidiabetic medications for weight loss among Lebanese adults and to identify sociodemographic, behavioural and health‐related factors associated with their use.

## 2 Methods

### 2.1 Study Design and Population

This cross‐sectional study assessed the KAP regarding the use of antidiabetic medications for weight loss among Lebanese adults. A self‐administered electronic questionnaire adapted from previously published studies was distributed to adults using a convenience sampling approach to facilitate rapid nationwide data collection. Data collection was conducted over a one‐month period, from June 20 to July 20, 2025, to allow sufficient time for participant recruitment and survey completion. The questionnaire was designed using an online survey platform and disseminated through links shared on multiple social media platforms, including Facebook, Instagram, Twitter and WhatsApp, to achieve broad national coverage. Participants were recruited using a non‐probability convenience sampling approach through social media platforms. Although this strategy facilitated rapid nationwide dissemination and participation, web‐based convenience sampling is inherently susceptible to selection bias because participation depends on internet access, digital literacy and voluntary engagement [18]. Consequently, individuals with higher educational attainment, greater health awareness and stronger interest in healthcare‐related topics may have been more likely to participate. Therefore, the resulting sample may not be fully representative of the Lebanese adult population.

Eligible participants were Lebanese residents aged 18 years or older who were aware of antidiabetic medications, regardless of prior use. Individuals younger than 18 years, non‐residents of Lebanon, and those with incomplete or inconsistent survey responses were excluded from the study. The sample size was calculated using the single‐population proportion formula, assuming *p* = 0.50, 95% confidence level, and 5% margin of error. A minimum sample of 377 participants was required, and this number was increased to 400 to account for potential incomplete responses. A total of 405 participants were ultimately included in the final analysis.

### 2.2 Data Collection

Data were collected through an anonymous online survey, which remained open for 1 month, with bi‐weekly reminders sent to enhance response rates. Participants were provided with detailed information regarding the study objectives, and electronic informed consent was obtained prior to survey initiation. All collected data were anonymized to ensure participant confidentiality.

The structured questionnaire was developed based on previously published studies and expert input and consisted of sections assessing sociodemographic characteristics, knowledge, attitudes and practices related to the use of antidiabetic medications for weight loss. The survey was administered in both Arabic and English. The questionnaire was primarily adapted from the study by Abutaima et al. [13], which evaluated KAP toward the use of antidiabetic medications such as Ozempic, Saxenda, Mounjaro and Glucophage for weight loss, with additional attitude and practice items adapted from the study by Algarni et al. [14].

The questionnaire was pilot‐tested among 30 participants who were not included in the final analysis to assess clarity and comprehensibility. Minor modifications were subsequently made based on participant feedback before dissemination of the final version. Questionnaires containing missing or inconsistent responses were excluded from the analysis.

Content validity of the questionnaire was assessed through expert review, during which questionnaire items were evaluated for relevance, clarity and suitability for the study objectives. The questionnaire was adapted from previously published instruments and underwent expert review and pilot testing prior to administration. Similar approaches have been employed in cross‐sectional KAP studies, where instrument adaptation and assessment of face and content validity are commonly used to support questionnaire suitability for the target population. Formal psychometric evaluation, including assessment of internal consistency reliability (Cronbach's alpha), exploratory or confirmatory factor analysis, and construct validity, was not performed as part of the present study. Consequently, although expert review and pilot testing supported face and content validity, the measurement properties of the knowledge and attitude scales cannot be fully established. Future studies should perform comprehensive psychometric validation to further evaluate the reliability and validity of the questionnaire in Lebanese populations.

### 2.3 Statistical Analysis

Data were analysed using IBM SPSS Statistics version 26 (IBM Corp., Armonk, NY, USA). Descriptive and inferential statistical methods were employed, with all statistical tests conducted as two‐tailed and a *p*‐value < 0.05 considered statistically significant.

Categorical variables were summarized using frequencies and percentages, whereas continuous variables were described using means ± standard deviations (SD), medians, minimum and maximum values, and percentile distributions.

Knowledge scores were calculated by assigning one point for each correct response and zero points for incorrect or ‘don't know’ responses. The total knowledge score was converted into a percentage score ranging from 0 to 100 using the formula: (obtained score/maximum possible score) × 100. Attitude scores were calculated by summing responses to the attitude items measured on a five‐point Likert scale after reverse coding negatively worded statements. The resulting scores were transformed to a 100‐point scale to facilitate interpretation and comparison.

For descriptive purposes, knowledge scores were categorized according to Bloom's cut‐off criteria as low (< 60%), moderate (60%–79%) and high (≥ 80%) knowledge levels [19]. Similarly, attitude scores were classified as low (< 60%), moderate (60%–79%) and high (≥ 80%) levels of appropriate attitudes toward the safe use of antidiabetic medications. However, all inferential statistical analyses were performed using the continuous knowledge and attitude scores to preserve statistical power and avoid information loss associated with categorization.

Bivariate analyses were conducted to examine associations between participant characteristics and study outcomes. Independent‐samples *t*‐tests and one‐way analysis of variance (ANOVA) were used to compare mean knowledge and attitude scores across sociodemographic and clinical characteristics. Pearson correlation coefficients were calculated to assess the relationship between knowledge and attitude scores. Associations between participant characteristics and the use of antidiabetic medications for weight loss were evaluated using chi‐squared tests for categorical variables and independent‐samples *t*‐tests for continuous variables.

Variables identified as statistically significant in the bivariate analyses were considered candidate variables for inclusion in the subsequent multivariable regression models. The bivariate analyses were used as an exploratory screening approach to identify factors potentially associated with the study outcomes. The primary conclusions of the study are based on the adjusted multivariable analyses, whereas findings from the bivariate analyses should be interpreted as exploratory. We acknowledge that variable selection based primarily on bivariate associations may not capture all theoretically relevant confounders and therefore may be subject to residual confounding.

For analytical purposes, education level was dichotomized into lower educational attainment (primary, secondary or bachelor level) and higher educational attainment (university or postgraduate degree). Chronic medication use was categorized as yes/no, and governorates were regrouped into Beirut/Mount Lebanon versus other regions.

Variables showing a statistically significant association in bivariate analyses (*p* < 0.05) were considered candidate variables for inclusion in the multivariable models. This exploratory approach was selected to identify factors potentially associated with the study outcomes. Two multiple linear regression models were constructed to identify factors independently associated with knowledge and attitude scores, respectively, while a binary logistic regression model was performed to identify factors independently associated with the use of antidiabetic medications for weight loss. Results from the linear regression analyses were reported using unstandardized regression coefficients (*B*) with corresponding 95% confidence intervals (CI), whereas logistic regression results were reported as adjusted odds ratios (aOR) with 95% CIs.

Given the exploratory nature of the study, no formal adjustment for multiple comparisons was applied; therefore, findings from bivariate analyses should be interpreted with caution. The primary inferential conclusions were based on the multivariable regression analyses.

Internal consistency reliability (e.g., Cronbach's alpha), construct validity, and exploratory or confirmatory factor analyses were not assessed, as the study focused on adapting previously published instruments and evaluating their face and content validity through expert review and pilot testing rather than conducting comprehensive psychometric validation.

### 2.4 Ethical Considerations

Ethical approval was obtained from the Lebanese Hospital Geitaoui–University Medical Center Institutional Review Board (IRB) (Approval Code: 2025‐IRB‐012; Approval Date: 19 March 2025) prior to the initiation of the study. Participants were informed about the study's purpose, procedures, potential risks and benefits, and electronic informed consent was obtained from all participants. Confidentiality and anonymity were strictly maintained throughout the study, and participants were informed of their right to withdraw at any time without any consequences. The study was conducted in accordance with the ethical principles outlined in the Declaration of Helsinki.

## 3 Results

### 3.1 Participant Characteristics

A total of 405 participants were included in the analysis. The mean age was 30.68 ± 8.48 years (range: 18–71), and most participants were female (65.4%). The majority were aged ≤ 30 years (61.7%) and had a high level of education, with 87.4% holding at least a university degree. More than half of the participants were single (65.2%), worked in medical‐related fields (57.0%), and were engaged in occupations involving physical labour (55.6%). Most respondents resided in Mount Lebanon (59.0%) or Beirut (27.2%), and 52.6% lived in urban areas. In addition, 72.8% reported having medical insurance. Detailed sociodemographic characteristics are presented in Table 1.

**TABLE 1:** Sociodemographic characteristics, clinical status, lifestyle behaviours and weight management practices of the study population (*N* = 405).

Variable	Category	*N* (%)
**Sociodemographic characteristics**		
Gender	Male	140 (34.6)
	Female	265 (65.4)
Age (in years)	Mean ± SD	30.68 ± 8.48
	Range	18–71
Age	≤ 30 years	250 (61.7)
	> 30 years	155 (38.3)
Education	Primary school or below	3 (0.7)
	High school/Technical secondary school	17 (4.2)
	Bachelor's degree	31 (7.7)
	University degree	194 (47.9)
	Postgraduate degree (Master's, Ph.D.)	160 (39.5)
Marital status	Single	264 (65.2)
	Married	134 (33.1)
	Divorced	5 (1.2)
	Widowed	2 (0.5)
Medical‐related occupation	No	174 (43.0)
	Yes	231 (57.0)
Physical labour occupation	No	180 (44.4)
	Yes	225 (55.6)
Monthly income (in $)	Less than 500$	128 (31.6)
	Between 500$ and 1500$	209 (51.6)
	Between 1500$ and 2500$	35 (8.6)
	More than 2500$	33 (8.1)
Living governorate	Beirut	110 (27.2)
	Mount Lebanon	239 (59.0)
	South Lebanon/Nabatiyeh	25 (6.2)
	Beqaa/Baalbek–Hermel	14 (3.5)
	North Lebanon/Akkar	17 (4.2)
Residence	Rural	192 (47.4)
	Urban	213 (52.6)
**Comorbidities and lifestyle**		
Medical insurance coverage	No	110 (27.2)
	Yes	295 (72.8)
What is your weight?	Mean ± SD	72.77 ± 16.75
	Range	42–135
What is your height?	Mean ± SD	167.65 ± 8.75
	Range	147–196
BMI	Mean ± SD	25.77 ± 4.99
	Range	16–46
Obesity	No	330 (81.5)
	Yes	75 (18.5)
Presence of chronic diseases	No	329 (81.2)
	Yes	76 (18.8)
Type of chronic diseases	Diabetes	15 (3.7)
	Hypertension	13 (3.2)
	Hypercholesterolemia	15 (3.7)
	Thyroid disease	11 (2.7)
	Cardiovascular disease	1 (0.2)
	Liver disease	1 (0.2)
	Other	36 (8.9)
Do you take chronic medications?	No	323 (79.8)
	1 medication	49 (12.1)
	2 medications	17 (4.2)
	3 medications	7 (1.7)
	More than 3 medications	9 (2.2)
Smoking	No	253 (62.5)
	Yes	152 (37.5)
**Lifestyle characteristics**		
Physical exercise rhythm	Almost never	164 (40.5)
	1–2 per week	159 (39.3)
	3–4 per week	67 (16.5)
	5 or more per week	15 (3.7)
Walking duration	None	77 (19.0)
	< 30 min	161 (39.8)
	30–60 min	124 (30.6)
	> 1 h	43 (10.6)
Job type	Sedentary	72 (17.8)
	Low activity	111 (27.4)
	Moderate activity	157 (38.8)
	High activity	65 (16.0)
**Screening and weight management practices**		
Did you ever perform a screening for excess visceral fat?	No	328 (81.0)
	Yes	77 (19.0)
Did you ever consult a dietitian?	No	207 (51.1)
	Yes	198 (48.9)
Do you follow a diet to lose weight?	Never	190 (46.9)
	Only once	75 (18.5)
	More than once	140 (34.6)
Did you ever encounter complications related to weight control?	No	241 (59.5)
	Yes	164 (40.5)
Did you ever undergo a surgery (bariatric surgery to lose weight)?	No	396 (97.8)
	Yes	9 (2.2)

Abbreviations: $, US dollar; BMI, body mass index; cm, centimetre; kg, kilogram; SD, standard deviation.

The mean BMI was 25.77 ± 4.99 kg/m^2^, and 18.5% of participants were classified as obese. Chronic diseases were reported by 18.8% of respondents, with diabetes mellitus and hypercholesterolemia each reported by 3.7%. The majority (79.8%) did not report the use of chronic medications. Smoking was reported by 37.5% of participants. With respect to lifestyle habits, 40.5% reported minimal physical activity, and 39.8% walked < 30 min per day. Only 31.6% reported currently following a weight‐loss diet, and 2.2% had previously undergone bariatric surgery.

The study sample was characterized by a relatively high educational level, with 87.4% of participants reporting university or postgraduate education and 57.0% employed in medical‐related fields. These characteristics should be considered when interpreting the findings, as they may be associated with differences in baseline health literacy and familiarity with medication‐related information.

### 3.2 Knowledge Regarding Antidiabetic Medications for Weight Loss

**FIGURE 1:**
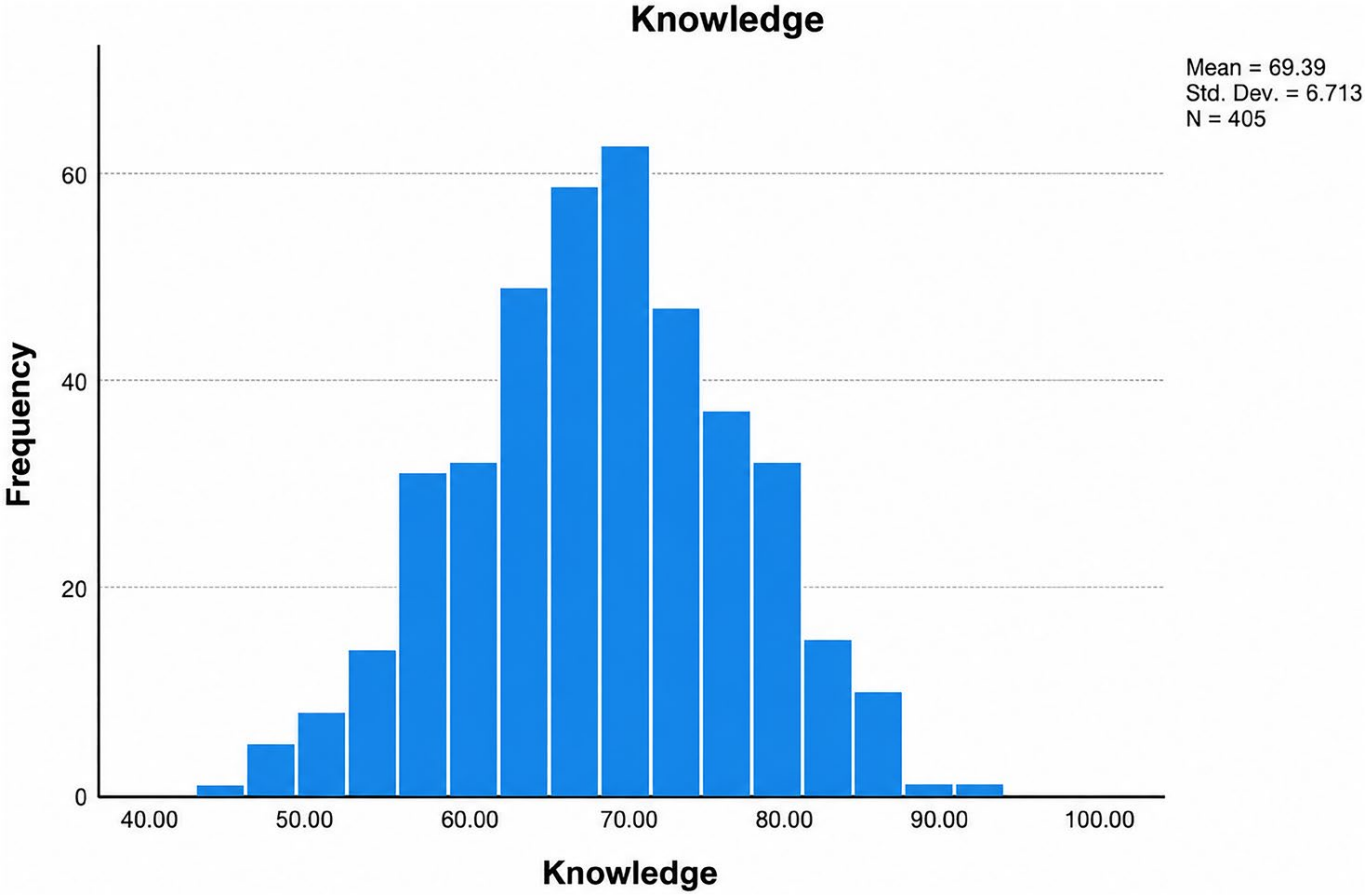
Knowledge score about antidiabetic medications for weight loss among the study population.

The mean knowledge score was 69.39 ± 8.71 out of 100, with scores ranging from 45.16 to 93.55. The median score was 70.97, while the interquartile range extended from 64.52 (25th percentile) to 74.19 (75th percentile). According to Bloom's cut‐off criteria, the overall mean score corresponded to a moderate level of knowledge. Furthermore, 80.0% of participants demonstrated moderate or high knowledge levels (Figure 1; Table S2).

The most commonly recognized medications were Ozempic (68.4%) and Glucophage (52.3%), followed by Saxenda (32.3%) and Mounjaro (28.6%). When asked to identify antidiabetic medications commonly perceived by participants as approved for weight management, 59.0% selected Ozempic, while smaller proportions identified Glucophage (33.8%) and Saxenda (23.7%). Because regulatory approval differs according to medication formulation, dosage, indication and jurisdiction, these responses should be interpreted as reflecting participants' knowledge rather than the actual regulatory approval status of individual products. Diet and physical exercise were correctly identified by most participants as effective weight‐loss strategies (92.3% and 90.1%, respectively). Nausea/vomiting (73.3%) and diarrhoea (48.4%) were the most frequently recognized side effects of injectable antidiabetic medications (Table S1).

Higher mean knowledge scores were observed among participants with university or postgraduate education compared with those with lower educational levels (*p* = 0.005), and among those working in medical‐related occupations compared with non‐medical occupations (*p* < 0.001). Knowledge scores were also higher among urban residents (*p* = 0.042) and non‐smokers (*p* = 0.031). Although participants who had consulted a dietitian (70.09 ± 8.11) or undergone visceral fat screening (71.05 ± 8.44) had slightly higher mean knowledge scores than those who had not (68.72 ± 9.22 and 69.00 ± 8.74, respectively), these differences were not statistically significant (*p* = 0.115 and *p* = 0.063, respectively). No significant differences were observed according to age, gender, marital status, income, physical labour occupation, medical insurance coverage, presence of chronic diseases, chronic medication use, physical activity, walking duration, job type or history of weight‐related complications (Table 2).

**TABLE 2:** Factors associated with knowledge about antidiabetic medications for weight loss among the study population.

Variable	Category	*N*	Mean ± SD	Range	*p*
Age	≤ 30 years	250	69.37 ± 8.16	48.39–87.10	0.946
	> 30 years	155	69.43 ± 9.56	45.16–93.55	
Gender	Male	140	69.38 ± 8.61	45.16–87.10	0.983
	Female	265	69.40 ± 8.79	48.39–93.55	
Education	Primary, secondary or Bachelor level	51	66.22 ± 9.79	45.16–93.55	0.005
	University or Postgraduate degree	354	69.85 ± 8.46	48.39–90.32	
Marital status	Married	134	68.83 ± 9.43	45.16–93.55	0.359
	Others	271	69.67 ± 8.34	48.39–90.32	
Medical‐related occupation	No	174	66.46 ± 8.26	45.16–87.10	< 0.001
	Yes	231	71.60 ± 8.41	51.61–93.55	
Physical labour occupation	No	180	69.70 ± 9.09	45.16–93.55	0.530
	Yes	225	69.15 ± 8.41	48.39–87.10	
Monthly income (in $)	Less than 500$	128	67.74 ± 8.29	45.16–83.87	0.010
	Between 500$ and 1500$	209	69.69 ± 8.88	48.39–93.55	
	Between 1500$ and 2500$	35	73.09 ± 7.74	58.06–87.10	
	More than 2500$	33	69.99 ± 9.11	51.61–90.32	
Living governorate	Beirut/Mount Lebanon	349	69.17 ± 8.65	45.16–93.55	0.213
	Others	56	70.74 ± 9.04	51.61–87.10	
Residence	Rural	192	68.46 ± 8.96	48.39–93.55	0.042
	Urban	213	70.23 ± 8.42	45.16–90.32	
Medical insurance coverage	No	110	68.48 ± 8.49	45.16–87.10	0.197
	Yes	295	69.73 ± 8.78	48.39–93.55	
Presence of chronic diseases	No	329	69.67 ± 8.60	45.16–93.55	0.174
	Yes	76	68.17 ± 9.15	48.39–83.87	
Do you take chronic medications?	No	323	69.36 ± 8.72	45.16–93.55	0.888
	Yes	82	69.51 ± 8.72	48.39–87.10	
Smoking	No	253	70.11 ± 8.87	48.39–93.55	0.031
	Yes	152	68.19 ± 8.34	45.16–90.32	
Did you ever perform a screening for excess visceral fat?	No	328	69.00 ± 8.74	45.16–93.55	0.063
	Yes	77	71.05 ± 8.44	48.39–87.10	
Did you ever consult a dietitian?	No	207	68.72 ± 9.22	45.16–93.55	0.115
	Yes	198	70.09 ± 8.11	48.39–90.32	
Do you follow a diet to lose weight?	Never	190	68.64 ± 9.51	45.16–93.55	0.227
	Only once	75	69.59 ± 8.08	48.39–87.10	
	More than once	140	70.30 ± 7.83	51.61–87.10	
Did you ever encounter complications related to weight control?	No	241	69.03 ± 8.90	45.16–93.55	0.309
	Yes	164	69.93 ± 8.43	48.39–87.10	
Did you ever undergo a surgery (bariatric surgery to lose weight)?	No	396	69.38 ± 8.73	45.16–93.55	0.862
	Yes	9	69.89 ± 8.38	58.06–83.87	
Physical exercise rhythm	Almost never	164	69.39 ± 9.29	48.39–93.55	0.995
	1–2 per week	159	69.28 ± 8.63	48.39–87.10	
	3–4 per week	67	69.57 ± 7.85	45.16–90.32	
	5 or more per week	15	69.68 ± 7.40	54.84–83.87	
Walking duration	None	77	68.71 ± 9.52	48.39–87.10	0.578
	< 30 min	161	68.98 ± 8.94	45.16–87.10	
	30–60 min	124	70.19 ± 8.41	48.39–93.55	
	> 1 h	43	69.84 ± 7.14	58.06–87.10	
Job type	Sedentary	72	68.82 ± 8.60	48.39–87.10	0.904
	Low activity	111	69.81 ± 9.00	48.39–87.10	
	Moderate activity	157	69.34 ± 8.81	45.16–93.55	
	High activity	65	69.43 ± 8.27	51.61–87.10	

*Note:* *p*‐values derived from independent *t*‐tests or one‐way ANOVA, as appropriate.

Abbreviation: SD, standard deviation.

Multiple linear regression analysis of factors associated with knowledge scores on antidiabetic medications for weight loss demonstrated that higher education and employment in a medical‐related occupation were significantly associated with greater knowledge. Specifically, higher education was positively associated with knowledge scores (*B* = 2.625, *p* = 0.037), and working in a medical‐related field showed a strong positive association (*B* = 4.893, *p* < 0.001). Other factors, including income, residence and smoking status, were not significantly associated with knowledge scores. Although higher income and urban residence exhibited slight positive trends, these were not statistically significant. Smoking showed a negative but non‐significant association. Overall, these findings suggest the association of educational attainment and professional background with the awareness of antidiabetic medications for weight management (Table 3).

**TABLE 3:** Multiple linear regression analysis of factors associated with knowledge scores regarding antidiabetic medications for weight loss.

Variable	*B*	SE	*p*	95% CI
Constant	66.463	0.633	< 0.001	65.219–67.706
Education	2.625	1.256	0.037	0.156–5.095
Medical‐related occupation	4.893	0.842	< 0.001	3.238–6.549

*Note:* Dependent Variable: Knowledge score regarding antidiabetic medications for weight loss.

Abbreviations: *B*, unstandardized regression coefficient; CI, confidence interval; SE, standard error.

### 3.3 Attitudes Toward the Use of Antidiabetic Medications for Weight Loss

The mean attitude score was 69.11 ± 5.54 out of 100, with scores ranging from 54.67 to 89.33. The median score was 69.33, and the interquartile range extended from 65.33 (25th percentile) to 72.00 (75th percentile). According to Bloom's cut‐off criteria, the overall mean score corresponded to a moderate level of appropriate attitude toward the safe use of antidiabetic medications for weight loss (Table S3).

A majority of participants disagreed that antidiabetic medications are safe and free of complications (76.8%) or that they are more effective than diet and exercise (74.8%). Most participants reported concerns regarding insufficient regulatory supervision of these medications in Lebanon (66.9%). Conversely, strong agreement was observed regarding the need for specialist counselling (92.3%) and medical supervision (69.2%) when using antidiabetic medications for weight management (Table 4).

**TABLE 4:** Attitudes toward the use of antidiabetic medications for weight loss among the study population.

		*N* (%)	Mean ± SD
There is sufficient supervision in Lebanon by the concerned authorities on the dispensing of antidiabetic medications used for weight‐loss purposes*	Strongly disagree	96 (23.7)	3.80 ± 0.94
	Disagree	175 (43.2)	
	Neutral	95 (23.5)	
	Agree	33 (8.1)	
	Strongly agree	6 (1.5)	
These medications can be used for weight loss, but under medical supervision	Strongly disagree	13 (3.2)	3.62 ± 1.01
	Disagree	62 (15.3)	
	Neutral	50 (12.3)	
	Agree	221 (54.6)	
	Strongly agree	59 (14.6)	
The use of these medications is the first and best option for weight loss*	Strongly disagree	97 (24.0)	3.83 ± 0.96
	Disagree	194 (47.9)	
	Neutral	70 (17.3)	
	Agree	36 (8.9)	
	Strongly agree	8 (2)	
These medications help you make the lifestyle changes you need to lose weight and improve your health	Strongly disagree	40 (9.9)	2.80 ± 1.07
	Disagree	141 (34.8)	
	Neutral	104 (25.7)	
	Agree	101 (24.9)	
	Strongly agree	19 (4.7)	
These medications are considered safe and can be used without complications*	Strongly disagree	102 (25.2)	3.93 ± 0.87
	Disagree	209 (51.6)	
	Neutral	61 (15.1)	
	Agree	31 (7.7)	
	Strongly agree	2 (0.5)	
The effectiveness of these medications for weight loss is guaranteed*	Strongly disagree	48 (11.9)	3.56 ± 0.09
	Disagree	191 (47.2)	
	Neutral	109 (26.9)	
	Agree	53 (13.1)	
	Strongly agree	4 (1.0)	
The efficacy of these medications is long‐lasting*	Strongly disagree	73 (18.0)	3.76 ± 0.87
	Disagree	200 (49.4)	
	Neutral	100 (24.7)	
	Agree	27 (6.7)	
	Strongly agree	5 (1.2)	
Weight Management Medications are more effective than diet/exercise*	Strongly disagree	115 (28.4)	3.85 ± 1.07
	Disagree	188 (46.4)	
	Neutral	40 (9.9)	
	Agree	49 (12.1)	
	Strongly agree	13 (3.2)	
Weight Management Medications should be restricted to those who failed to lose weight with diet/exercise	Strongly disagree	18 (4.4)	3.48 ± 1.03
	Disagree	63 (15.6)	
	Neutral	75 (18.5)	
	Agree	203 (50.1)	
	Strongly agree	46 (11.4)	
Weight Management Medications are safe	Strongly disagree	88 (21.7)	2.11 ± 0.85
	Disagree	221 (54.6)	
	Neutral	65 (16.0)	
	Agree	27 (6.7)	
	Strongly agree	4 (1.0)	
Weight Management Medications require a specialist's counselling	Strongly disagree	2 (0.5)	4.44 ± 0.71
	Disagree	5 (1.2)	
	Neutral	24 (5.9)	
	Agree	156 (38.5)	
	Strongly agree	218 (53.8)	
Weight Management Medications are more convenient to use	Strongly disagree	20 (4.9)	3.25 ± 1.05
	Disagree	95 (23.5)	
	Neutral	84 (20.7)	
	Agree	176 (43.5)	
	Strongly agree	30 (7.4)	
Weight Management Medications enable rapid results	Strongly disagree	16 (4.0)	3.25 ± 0.98
	Disagree	85 (21.0)	
	Neutral	110 (27.2)	
	Agree	171 (42.2)	
	Strongly agree	23 (5.7)	
Weight loss with Weight Management Medications is long‐lasting*	Strongly disagree	56 (13.8)	3.68 ± 0.87
	Disagree	207 (51.1)	
	Neutral	103 (25.4)	
	Agree	33 (8.1)	
	Strongly agree	6 (1.5)	
Weight Management Medications should only be for people with extreme obesity*	Strongly disagree	13 (3.2)	2.49 ± 1.01
	Disagree	65 (16.0)	
	Neutral	80 (19.8)	
	Agree	195 (48.1)	
	Strongly agree	52 (12.8)	

*Note:* Responses were rated on a 5‐point Likert scale (1 = strongly disagree to 5 = strongly agree). Items marked with * were negatively worded and reverse‐coded for attitude score calculation. Higher scores indicate more appropriate attitudes toward the safe and medically supervised use of antidiabetic medications for weight loss, including recognition of the importance of medical supervision, specialist counselling and regulatory oversight.

Higher attitude scores were observed among participants with university or postgraduate education (*p* = 0.001), those working in medical‐related occupations (*p* < 0.001), urban residents (*p* = 0.035), non‐smokers (*p* = 0.037), participants following a weight‐loss diet (*p* = 0.018), and those who reported experiencing complications related to weight control (*p* = 0.001). Consulting a dietitian (69.14 ± 5.52 vs. 69.08 ± 5.56; *p* = 0.919) or undergoing visceral fat screening (69.56 ± 5.92 vs. 69.00 ± 5.45; *p* = 0.429) resulted in slightly higher scores, though these differences were not statistically significant. No significant differences were found according to age, gender, marital status, monthly income, physical labour, medical insurance coverage, chronic diseases, chronic medication use, physical activity patterns, walking duration, job type or history of bariatric surgery (Table 5).

**TABLE 5:** Factors associated with attitudes toward the usage of antidiabetic medications for weight loss among the study population.

Variable	Category	*N*	Mean ± SD	Range	*p*
Age	≤ 30 years	250	69.39 ± 5.65	54.67–89.33	0.201
	> 30 years	155	68.66 ± 5.34	56.00–89.33	
Gender	Male	140	68.59 ± 5.26	54.67–78.67	0.171
	Female	265	69.38 ± 5.67	54.67–89.33	
Education	Primary, secondary or Bachelor	51	66.80 ± 5.14	56.00–76.00	0.001
	University or Postgraduate	354	69.44 ± 5.52	54.67–89.33	
Marital status	Married	134	69.34 ± 5.37	54.67–88.00	0.550
	Others	271	68.99 ± 5.62	54.67–89.33	
Medical‐related occupation	No	174	67.60 ± 5.56	54.67–89.33	< 0.001
	Yes	231	70.25 ± 5.25	54.67–89.33	
Physical labour occupation	No	180	68.85 ± 5.62	56.00–89.33	0.403
	Yes	225	69.32 ± 5.47	54.67–89.33	
Monthly income (in $)	Less than 500$	128	68.21 ± 5.34	54.67–84.00	0.165
	Between 500$ and 1500$	209	69.49 ± 5.70	54.67–89.33	
	Between 1500$ and 2500$	35	69.41 ± 5.39	61.33–82.67	
	More than 2500$	33	69.86 ± 5.13	58.67–78.67	
Living governorate	Beirut/Mount Lebanon	349	69.14 ± 5.59	54.67–89.33	0.794
	Others	56	68.93 ± 5.21	58.67–82.67	
Residence	Rural	192	68.50 ± 5.33	54.67–89.33	0.035
	Urban	213	69.66 ± 5.67	54.67–89.33	
Medical insurance coverage	No	110	68.93 ± 6.27	56.00–89.33	0.697
	Yes	295	69.18 ± 5.25	54.67–88.00	
Presence of chronic diseases	No	329	69.20 ± 5.68	54.67–89.33	0.477
	Yes	76	68.70 ± 4.88	54.67–84.00	
Do you take chronic medications?	No	323	69.10 ± 5.67	54.67–89.33	0.958
	Yes	82	69.14 ± 5.01	54.67–80.00	
Smoking	No	253	69.55 ± 5.25	58.67–89.33	0.037
	Yes	152	68.37 ± 5.93	54.67–89.33	
Did you ever perform a screening for excess visceral fat?	No	328	69.00 ± 5.45	54.67–88.00	0.429
	Yes	77	69.56 ± 5.92	58.67–89.33	
Did you ever consult a dietitian?	No	207	69.08 ± 5.56	56.00–88.00	0.919
	Yes	198	69.14 ± 5.52	54.67–89.33	
Do you follow a diet to lose weight?	Never	190	68.29 ± 5.61	54.67–88.00	0.018
	Only once	75	69.69 ± 5.49	54.67–85.33	
	More than once	140	69.91 ± 5.34	54.67–89.33	
Did you ever encounter complications related to weight control?	No	241	68.38 ± 5.46	54.67–88.00	0.001
	Yes	164	70.18 ± 5.49	54.67–89.33	
Did you ever undergo surgery (bariatric surgery to lose weight)?	No	396	69.05 ± 5.37	54.67–88.00	0.133
	Yes	9	71.85 ± 10.74	58.67–89.33	
Physical exercise rhythm	Almost never	164	69.26 ± 5.25	54.67–89.33	0.304
	1–2 per week	159	68.68 ± 5.51	54.67–85.33	
	3–4 per week	67	70.03 ± 6.16	56.00–89.33	
	5 or more per week	15	67.91 ± 5.78	58.67–77.33	
Walking duration	None	77	67.77 ± 4.49	58.67–78.67	0.098
	< 30 min	161	69.18 ± 5.78	54.67–89.33	
	30–60 min	124	69.57 ± 5.65	54.67–89.33	
	> 1 h	43	69.92 ± 5.75	60.00–80.00	
Job type	Sedentary	72	68.46 ± 5.06	58.67–82.67	0.139
	Low activity	111	69.00 ± 5.92	54.67–89.33	
	Moderate activity	157	69.85 ± 5.12	56.00–88.00	
	High activity	65	68.23 ± 6.19	54.67–89.33	

*Note:* *p*‐values were derived from independent‐samples *t*‐tests or one‐way analysis of variance (ANOVA), as appropriate.

In multiple linear regression analysis, higher education level (*B* = 2.016, *p* = 0.012), medical‐related occupation (*B* = 2.418, *p* < 0.001), and a history of weight‐control complications (*B* = 1.661, *p* = 0.002) were independently associated with more appropriate attitudes toward the safe use of antidiabetic medications for weight loss. However, factors such as residence, smoking status and diet adherence did not significantly affect attitude scores. These findings suggest that education, professional exposure and personal experiences with weight‐related challenges are associated with attitudes toward antidiabetic medications (Table 6).

**TABLE 6:** Multiple linear regression analysis of factors associated with attitudes toward the use of antidiabetic medications for weight loss.

Variable	*B*	SE	*p*	95% CI
(Constant)	63.280	1.517	< 0.001	60.298–66.263
Education	2.016	0.801	0.012	0.441–3.590
Medical‐related occupation	2.418	0.536	< 0.001	1.364–3.472
Did you ever encounter complications related to weight control?	1.661	0.536	0.002	0.606–2.715

*Note:* Dependent variable: Attitude toward the use of antidiabetic medications used for weight loss.

Abbreviations: *B*, unstandardized regression coefficient; CI, confidence interval; SE, standard error.

### 3.4 Use of Antidiabetic Medications for Weight Loss

Overall, 12.3% (*n* = 50) of participants reported having used antidiabetic medications for weight loss. Among users, Ozempic was the most frequently reported medication (64.0%), followed by Glucophage (26.0%). Approximately 54.0% of users reported a weight loss of < 10 kg, while 46.0% reported a weight loss of 10 kg or more. Weight regain after discontinuation was reported by 46.0% of users. Prior medical consultation was reported by 68.0% of users, and 62.0% experienced side effects during use (Table 7).

**TABLE 7:** Patterns and characteristics of antidiabetic medication use for weight loss among study participants.

Variable	Category	*N* (%)
Have you used any medication for weight‐loss purposes in the last 12 months?	No	355 (87.7)
	Yes	50 (12.3)
Which of the following antidiabetic medications have you used for weight‐loss purposes?	Glucophage	13 (26.0)
	Mounjaro	3 (6.0)
	Ozempic	32 (64.0)
	Saxenda	2 (4.0)
What other ways do you usually follow to lose weight in addition to using antidiabetic medications?	Diet	32 (7.9)
	Exercising	19 (4.7)
	Herbal products	4 (1.0)
	Other prescription medications	3 (0.7)
	Others	8 (2.0)
What is the source of these medications?	Pharmacy	43 (10.6)
	Hospitals	2 (0.5)
	Facebook Pages	3 (0.7)
	Private clinics/medical centres	3 (0.7)
	Family and friends abroad	1 (0.2)
Have you had difficulty obtaining these medications recently?	No	38 (76.0)
	Yes	12 (24.0)
If you have ever used these medications, how much weight (kg) did you lose?	< 10 kg	27 (54.0)
	≥ 10 kg	23 (46.0)
Do you check the source of these medications when purchasing them?	No	17 (34.0)
	Yes	33 (66.0)
Did you consult a doctor before using these medications?	No	16 (32.0)
	Yes	34 (68.0)
Did you gain any weight back after you stopped using these medications?	No	27 (54.0)
	Yes	23 (46.0)
Have you experienced any side effects when using these medicines?	No	19 (38.0)
	Yes	31 (62.0)
Do you read medical information about the products you use to lose weight?	No	16 (32.0)
	Yes	34 (68.0)

*Note:* Percentages are calculated based on the total sample (*N* = 405) unless otherwise specified. Items related to medication use characteristics were calculated among users only (*n* = 50). Ozempic (semaglutide), Saxenda (liraglutide), Mounjaro (tirzepatide), Glucophage (metformin).

### 3.5 Association Between Knowledge, Attitudes and Medication Use

**FIGURE 2:**
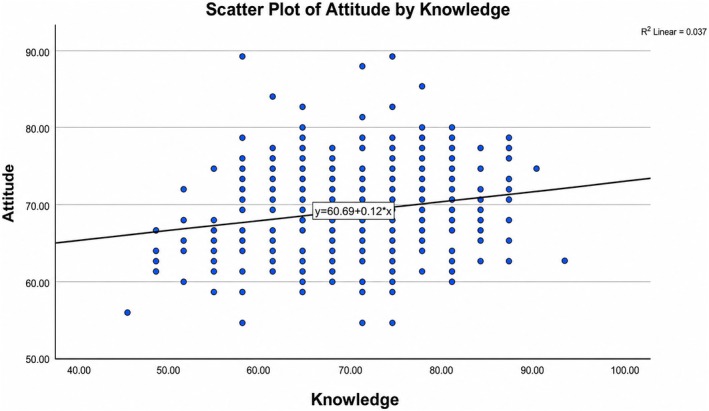
Scatter plot for the correlation between knowledge and attitudes toward antidiabetic medications for weight loss.

A weak but statistically significant positive correlation was observed between knowledge and attitude scores (*r* = 0.191, *p* < 0.001) (Figure 2).

In bivariate analyses, the use of antidiabetic medications for weight loss was more frequent among participants aged over 30 years (*p* = 0.006), those with chronic diseases (*p* = 0.003), those taking chronic medications (*p* = 0.027), smokers (*p* = 0.024), participants who had followed a weight‐loss diet (*p* < 0.001), those who reported weight‐control complications (*p* < 0.001), individuals who had undergone visceral fat screening (*p* = 0.012), and those who had consulted a dietitian (*p* < 0.001). Lower use was observed among participants engaged in higher‐activity occupations (*p* = 0.026). No significant associations were observed with gender, education level, marital status, income, medical insurance, residence or physical activity level. Surgical history and physical activity levels were also not associated with medication use. Older age, chronic conditions and dieting history were significantly associated with medication use (Table 8).

**TABLE 8:** Bivariate associations between participant characteristics and the use of antidiabetic medications for weight loss.

Variable	Category	Have you used any medication for weight loss purposes in the last 12 months?			*p*
		No, *n* (%)	Yes, *n* (%)	Total, *n* (%)	
Age	≤ 30 years	228 (64.2)	22 (44.0)	250 (61.7)	0.006
	> 30 years	127 (35.8)	28 (56.0)	155 (38.3)	
Gender	Male	126 (35.5)	14 (28.0)	140 (34.6)	0.297
	Female	229 (64.5)	36 (72.0)	265 (65.4)	
Education	Primary, secondary or Bachelor	47 (13.2)	4 (8.0)	51 (12.6)	0.296
	University or Postgraduate	308 (86.8)	46 (92.0)	354 (87.4)	
Marital status	Married	114 (32.1)	20 (40.0)	134 (33.1)	0.267
	Others	241 (67.9)	30 (60.0)	271 (66.9)	
Medical‐related occupation	No	147 (41.4)	27 (54.0)	174 (43.0)	0.092
	Yes	208 (58.6)	23 (46.0)	231 (57.0)	
Physical labour occupation	No	155 (43.7)	25 (50.0)	180 (44.4)	0.398
	Yes	200 (56.3)	25 (50.0)	225 (55.6)	
Monthly income (in $)	Less than 500$	115 (32.4)	13 (26.0)	128 (31.6)	0.157
	Between 500$ and 1500$	186 (52.4)	23 (46.0)	209 (51.6)	
	Between 1500$ and 2500$	28 (7.9)	7 (14.0)	35 (8.6)	
	More than 2500$	26 (7.3)	7 (14.0)	33 (8.1)	
Living governorate	Beirut/Mount Lebanon	307 (86.5)	42 (84.0)	349 (86.2)	0.634
	Others	48 (13.5)	8 (16.0)	56 (13.8)	
Residence	Rural	172 (48.5)	20 (40.0)	192 (47.4)	0.263
	Urban	183 (51.5)	30 (60.0)	213 (52.6)	
Medical insurance coverage	No	98 (27.6)	12 (24.0)	110 (27.2)	0.592
	Yes	257 (72.4)	38 (76.0)	295 (72.8)	
Presence of chronic diseases	No	296 (83.4)	33 (66.0)	329 (81.2)	0.003
	Yes	59 (16.6)	17 (34.0)	76 (18.8)	
Do you take chronic medications?	No	289 (81.4)	34 (68.0)	323 (79.8)	0.027
	Yes	66 (18.6)	16 (32.0)	82 (20.2)	
Smoking	No	229 (64.5)	24 (48.0)	253 (62.5)	0.024
	Yes	126 (35.5)	26 (52.0)	152 (37.5)	
Did you ever perform a screening for excess visceral fat?	No	294 (82.8)	34 (68.0)	328 (81.0)	0.012
	Yes	61 (17.2)	16 (32.0)	77 (19.0)	
Did you ever consult a dietitian?	No	197 (55.5)	10 (20.0)	207 (51.1)	< 0.001
	Yes	158 (44.5)	40 (80.0)	198 (48.9)	
Do you follow a diet to lose weight?	Never	185 (52.1)	5 (10.0)	190 (46.9)	< 0.001
	Only once	66 (18.6)	9 (18.0)	75 (18.5)	
	More than once	104 (29.3)	36 (72.0)	140 (34.6)	
Did you ever encounter complications related to weight control?	No	235 (66.2)	6 (12.0)	241 (59.5)	< 0.001
	Yes	120 (33.8)	44 (88.0)	164 (40.5)	
Did you ever undergo a surgery (bariatric surgery to lose weight)?	No	348 (98.0)	48 (96.0)	396 (97.8)	0.362
	Yes	7 (2.0)	2 (4.0)	9 (2.2)	
Physical exercise rhythm	Almost never	141 (39.7)	23 (46.0)	164 (40.5)	0.434
	1–2 per week	140 (39.4)	19 (38.0)	159 (39.3)	
	3–4 per week	62 (17.5)	5 (10.0)	67 (16.5)	
	5 or more per week	12 (3.4)	3 (6.0)	15 (3.7)	
Walking duration	None	63 (17.7)	14 (28.0)	77 (19.0)	0.155
	< 30 min	140 (39.4)	21 (42.0)	161 (39.8)	
	30–60 min	111 (31.3)	13 (26.0)	124 (30.6)	
	> 1 h	41 (11.5)	2 (4.0)	43 (10.6)	
Job type	Sedentary	59 (16.6)	13 (26.0)	72 (17.8)	0.026
	Low activity	92 (25.9)	19 (38.0)	111 (27.4)	
	Moderate activity	142 (40.0)	15 (30.0)	157 (38.8)	
	High activity	62 (17.5)	3 (6.0)	65 (16.0)	

*Note:* *p*‐values were obtained using independent‐samples *t*‐tests or chi‐squared tests, as appropriate.

In bivariate analyses, the use of antidiabetic medications for weight loss was more frequent among older participants, smokers, individuals with chronic conditions, repeated dieting attempts, prior weight‐control complications, visceral fat screening and dietitian consultation, while lower use was observed among those with more physically active occupations (Table 8). Variables significant in bivariate analysis were subsequently included in the multivariable logistic regression model. Participants who reported using antidiabetic medications for weight loss had higher mean knowledge scores compared to non‐users (*p* = 0.047). No significant difference in attitude scores was observed between users and non‐users (*p* = 0.209).

In binary logistic regression analysis, smoking (OR = 3.13, 95% CI: 1.53–6.38, *p* = 0.002), experiencing weight‐control complications (OR = 8.50, 95% CI: 3.27–22.12, *p* < 0.001), adherence to a weight‐loss diet (OR = 2.21, 95% CI: 1.35–3.60, *p* = 0.002), and higher knowledge scores (OR = 1.05, 95% CI: 1.00–1.09, *p* = 0.036) were independently associated with the use of antidiabetic medications for weight loss. Participants engaged in more physically active jobs were less likely to use these medications (OR = 0.58, 95% CI: 0.41–0.84, *p* = 0.003).

Conversely, age, presence of chronic disease, use of chronic medications, visceral fat screening and dietitian consultation were not significantly associated with medication use (*p* > 0.05). Overall, these results suggest that behavioural and experiential factors, specifically smoking, weight‐control complications, dieting behaviour, job activity level and knowledge, are strongly associated with the use of antidiabetic medications for weight loss (Table 9).

**TABLE 9:** Multivariable binary logistic regression analysis of factors associated with the use of antidiabetic medications for weight loss.

Variable	aOR	95% CI	*p*
Smoking	3.13	1.53–6.38	0.002
Do you follow a diet to lose weight?	2.01	1.35–3.60	0.002
Did you ever encounter complications related to weight control?	8.50	3.27–22.12	< 0.001
Job type	0.58	0.41–0.84	0.003
Knowledge	1.05	1.00–1.09	0.036

*Note:* Variables with clinical relevance and/or significant associations in bivariate analyses (*p* < 0.05) were included in the multivariable logistic regression model. Results are presented as adjusted odds ratios (aOR) with 95% confidence intervals. Reference categories were non‐smoker, no diet, no previous weight‐control complications, non‐medical occupation and lowest knowledge score.

Abbreviations: aOR, adjusted odds ratio; CI, confidence interval.

In multivariable logistic regression analysis (Table 9), smoking was associated with a threefold increase in the odds of using antidiabetic medications for weight loss (aOR 3.13, 95% CI 1.53–6.38). Participants reporting prior weight‐control complications had substantially higher odds of use (aOR 8.50, 95% CI 3.27–22.12). Adherence to a weight‐loss diet (aOR 2.21, 95% CI 1.35–3.60) and higher knowledge scores (aOR 1.05 per unit increase, 95% CI 1.00–1.09) were also independently associated with use, whereas greater occupational physical activity was inversely associated (aOR 0.58, 95% CI 0.41–0.84).

## 4 Discussion

This nationwide study provides one of the first comprehensive assessments of KAP regarding antidiabetic medications used for weight loss among Lebanese adults. Overall, participants demonstrated moderate levels of knowledge and generally appropriate attitudes toward the safe use of these medications, while only a minority reported using them for weight loss. Most respondents relied primarily on lifestyle‐based strategies, such as dietary modification and physical activity, highlighting awareness of the importance of lifestyle interventions as the foundation of weight management despite growing interest in pharmacological approaches. Compared with available national demographic estimates, our study population included a substantially higher proportion of participants with university or postgraduate education and healthcare‐related occupations. This imbalance likely reflects the use of web‐based convenience sampling and may have resulted in overestimation of knowledge levels and more appropriate attitudes toward medication use. Consistent with this interpretation, higher educational attainment and employment in healthcare‐related occupations remained independent predictors of both higher knowledge and more appropriate attitudes in the multivariable analyses.

The study population consisted predominantly of young adults with a high educational level, with 87.4% reporting university or postgraduate education, and more than half working in medical‐related fields. This educational profile appears higher than educational attainment estimates reported for the general Lebanese population according to data from the UNESCO Institute for Statistics and the World Bank [20, 21]. Similarly, the proportion of respondents employed in medical‐related professions was substantially higher than would be expected in the general population, likely reflecting the online convenience sampling strategy and dissemination through social media platforms. Individuals with higher educational attainment generally demonstrate greater health literacy, improved access to healthcare information, and increased engagement in preventive health behaviours [22]. This interpretation is supported by our multivariable analyses, which identified both higher educational attainment and employment in medical‐related occupations as independent predictors of greater knowledge and more appropriate attitudes toward the safe use of antidiabetic medications for weight loss. Consequently, the knowledge levels and attitudes observed in this study may overestimate those present in the broader Lebanese population, and caution is warranted when generalizing these findings to all Lebanese adults.

The mean BMI of participants placed the average respondent in the overweight category, while fewer than one‐fifth were classified as obese. Reported comorbidities included diabetes mellitus and hypercholesterolemia, consistent with previous Lebanese epidemiological data [4, 23]. These findings highlight the coexistence of excess weight and cardiometabolic risk factors within the studied population and reinforce the growing importance of effective weight management strategies in Lebanon.

Physical activity levels were generally low, with many participants reporting little or no regular exercise and low occupational physical activity. These findings are consistent with previous Lebanese studies documenting declining physical activity levels and increasingly sedentary lifestyles, which may contribute to the high prevalence of overweight status observed in the population [24, 25]. Furthermore, engagement with structured weight management approaches, including consultations with dietitians, visceral fat assessment or bariatric surgery, was relatively limited. Collectively, these observations suggest suboptimal adoption of evidence‐based weight management strategies and highlight opportunities for strengthening preventive interventions targeting obesity and related chronic non‐communicable diseases such as hypertension, dyslipidemia and cardiovascular disease [26].

Participants demonstrated a moderate level of knowledge regarding the use of antidiabetic medications for weight loss. Awareness was highest for commonly recognized medications such as Ozempic and Glucophage, as well as for pharmacological options approved for weight management in appropriate formulations and regulatory jurisdictions, likely reflecting increasing exposure through healthcare settings, social media and digital platforms. Nevertheless, significant knowledge gaps remained regarding approved indications and safety profiles. While most participants recognized common gastrointestinal adverse effects, awareness of less frequently discussed but clinically significant risks, including pancreatitis, gallbladder disease, kidney impairment and potential thyroid tumours, was limited. Insufficient awareness of these risks may contribute to underestimation of potential harms associated with unsupervised medication use, underscoring the importance of physician‐led counselling and patient education. Furthermore, limited recognition of the multifactorial nature of obesity management may indicate the need for broader public education regarding comprehensive treatment approaches. These findings are consistent with regional and international studies reporting moderate but incomplete public knowledge regarding antidiabetic medications used for weight loss [14, 15, 16].

This study highlights the association of educational background, occupational exposure, and personal experiences with weight management, and participants' knowledge, attitudes, and use of antidiabetic medications for weight loss in Lebanon. Higher educational attainment and employment in medical‐related fields were associated with greater knowledge and more appropriate attitudes toward antidiabetic medications for weight loss. These findings are consistent with previous studies conducted in Saudi Arabia and Jordan [13, 15]. This may reflect higher health literacy, greater access to evidence‐based information, and increased familiarity with clinical practice and obesity pharmacotherapy. Collectively, these findings suggest that educational and professional exposure may influence health‐related decision‐making and perceptions regarding pharmacological interventions for weight management.

A valuable finding was the positive association between higher knowledge scores and the use of antidiabetic medications for weight loss. One possible explanation is that individuals who had already used or seriously considered using these medications actively sought information from healthcare professionals, scientific sources, product information leaflets or online resources. Consequently, higher knowledge may reflect increased exposure to medication‐related information rather than knowledge itself promoting medication use. Similarly, this association may indicate more informed healthcare‐seeking behaviour among some participants. Because of the cross‐sectional design, however, the temporal direction of this relationship cannot be determined.

Participants generally exhibited cautious attitudes toward the use of antidiabetic medications for weight loss. Most respondents expressed concerns regarding medication safety, regulatory oversight and the effectiveness of pharmacological therapy compared with lifestyle modification. In addition, many believed that these medications should be prescribed under medical supervision and reserved for individuals with severe obesity. Individuals who had previously experienced difficulties achieving weight loss through conventional methods showed more receptive to pharmacological options, possibly reflecting greater openness to medical intervention after unsuccessful lifestyle‐based attempts. Despite recognizing the potential effectiveness of pharmacological interventions, lifestyle modification remained the preferred approach to weight management among most participants. These findings are consistent with previous regional studies reporting concerns regarding medication safety, appropriate prescribing, and the role of pharmacological therapy as an adjunct rather than a substitute for lifestyle‐based interventions [13, 15].

Despite the generally cautious attitudes observed, attitudes were not independently associated with medication use. This finding may suggest that concerns regarding safety, regulation and appropriate prescribing do not always translate into avoidance of these medications, particularly among individuals who perceive potential benefits or have experienced difficulties with weight management. Alternatively, attitudes may represent general perceptions toward these medications rather than actual behavioural intentions or practices. Other contextual factors, including exposure to healthcare providers, social media discussions and medication‐related information sources, may contribute to the observed relationship between attitudes and medication use. This interpretation is consistent with behavioural theories suggesting that appropriate or inappropriate attitudes do not necessarily translate into behaviour, as decisions are often influenced by practical, social and environmental factors beyond individual perceptions [27, 28].

Several behavioural, socioeconomic and experiential factors were associated with the use of antidiabetic medications for weight loss. Smoking status, adherence to dietary interventions, previous weight‐related complications, occupational physical activity and higher knowledge scores were independently associated with medication use. However, these findings should be interpreted as correlational associations rather than causal relationships, given the cross‐sectional design of the study. The association between previous weight‐control complications and medication use warrants cautious interpretation. Individuals who reported difficulties achieving weight loss through conventional approaches may have been more likely to consider pharmacological therapies; alternatively, individuals using these medications may have had greater prior exposure to weight management challenges, and reverse causation cannot be excluded. Furthermore, residual confounding from unmeasured factors, such as obesity severity, healthcare access, psychological characteristics or motivation toward weight management, cannot be excluded and may partly explain the observed associations.

Similarly, the associations between smoking status, dietary adherence and medication use should not be interpreted as direct causal effects. These relationships may instead reflect underlying weight management behaviours, health‐related practices or greater engagement in strategies aimed at controlling body weight. Previous studies have linked smoking with body‐image concerns and weight‐control behaviours [29], while dietary adherence has been associated with broader engagement in weight management efforts [30]. Therefore, further longitudinal studies are needed to clarify the temporal relationships and underlying mechanisms linking these behavioural factors with the use of antidiabetic medications for weight loss.

An notable finding was the positive association between higher knowledge scores and the use of antidiabetic medications for weight loss. Although greater knowledge is often assumed to promote healthier decision‐making, this relationship may instead reflect increased awareness and familiarity with available pharmacological options. Individuals with higher knowledge levels may also have been more frequently exposed to medication‐related information through healthcare settings, professional environments, personal research or social media, thereby increasing familiarity with available pharmacological options. Such exposure may contribute to greater awareness of these therapies and may partly explain the observed association between higher knowledge scores and medication use. Alternatively, reverse causation cannot be excluded, as previous medication users may have acquired additional knowledge through their treatment experiences. Consequently, the observed association should not be interpreted as evidence that greater knowledge directly promotes medication use. Given the cross‐sectional design of the study, causal relationships between knowledge, attitudes and medication use cannot be established, and these findings should be interpreted as correlational and with caution. This pattern suggests that behavioural, socioeconomic and experiential factors may play a significant role in shaping public perceptions and practices regarding antidiabetic medications for weight loss in Lebanon.

Although only a minority of participants reported using antidiabetic medications for weight loss, the observed prevalence remains clinically relevant because even relatively infrequent unsupervised use may have important implications for medication safety and public health. Ozempic was the most commonly used medication, and pharmacies represented the primary source of acquisition. Use was frequently reported in combination with lifestyle modification or other weight management approaches, consistent with findings from previous regional studies [15]. However, a notable proportion of participants reported obtaining these medications through informal or non‐medical channels, raising concerns regarding inappropriate access and potential medication misuse. The relatively high proportion of participants reporting adverse effects is generally consistent with the well‐established gastrointestinal adverse‐event profile of GLP‐1 RAs reported in randomized clinical trials and real‐world observational studies [7, 8]. Although the present study did not assess the severity or specific types of adverse events, these findings reinforce the importance of physician‐guided treatment initiation, patient education regarding expected adverse effects, and regular clinical follow‐up.

Among medication users, weight loss was commonly reported, suggesting perceived effectiveness of these agents in supporting weight reduction. Although most participants reported consulting a physician before initiating treatment, nearly one‐third reported use without prior medical consultation. These findings are consistent with regional studies demonstrating frequent self‐directed use and limited professional oversight among individuals using GLP‐1 RAs and other antidiabetic medications for weight loss [13, 15]. Together, these findings suggest concerns regarding self‐directed medication use, inadequate medical supervision, and the need for improved monitoring of prescribing and dispensing practices. They also highlight the importance of physician‐guided prescribing, pharmacist‐led counselling, and patient education to promote the safe and appropriate use of these medications in Lebanon.

Nearly half of participants who discontinued treatment reported regaining weight, which is consistent with evidence demonstrating that weight regain commonly occurs after withdrawal of GLP‐1 RAs and other medications for weight management [31, 32]. These findings reinforce the understanding of obesity as a chronic relapsing condition requiring long‐term management rather than a short‐term intervention. They also highlight that pharmacological therapy should be integrated with sustainable lifestyle modifications, including dietary changes, regular physical activity and behavioural support, to optimize long‐term outcomes and reduce the likelihood of weight regain. Furthermore, these findings emphasize the importance of appropriate patient counselling before treatment initiation, including discussions regarding the potential for weight regain after discontinuation, the need for maintenance strategies when appropriate, and the importance of continued monitoring to support sustained treatment benefits.

Taken together, these findings underscore the importance of integrated strategies for weight management that combine education, medical supervision and lifestyle interventions. While pharmacological options may support individuals with persistent obesity, their safe and effective use requires appropriate guidance and regulatory oversight. The observed limited consultation with specialists (68%) and high prevalence of side effects (62%) further emphasize the need for targeted educational interventions and stronger regulatory control to ensure appropriate use, minimize adverse effects and optimize long‐term outcomes.

These findings provide important public health insights into the use of antidiabetic medications for weight management in Lebanon. The observed association between medication use and higher knowledge levels, previous weight‐related complications, smoking status and dietary adherence suggests that pharmacological weight management strategies may be more commonly considered among individuals who are actively engaged in weight‐control efforts or who have experienced challenges achieving weight loss through conventional approaches. However, these associations should be interpreted cautiously given the cross‐sectional design of the study.

The reported use of these medications without prior physician consultation, together with the considerable proportion of participants experiencing adverse effects and weight regain following treatment discontinuation, highlights the need for targeted patient education, physician‐guided prescribing and structured follow‐up. Educational initiatives should focus not only on increasing awareness of available therapies but also on addressing the specific challenges identified in this study. In particular, efforts should emphasize the importance of medical supervision before treatment initiation, counselling regarding expected adverse effects and appropriate monitoring, and realistic expectations about the long‐term management of obesity, including the potential for weight regain following treatment discontinuation.

Furthermore, the relationship between medication use and previous weight‐related complications emphasizes the role of healthcare professionals in identifying individuals who may benefit from pharmacological interventions and ensuring appropriate monitoring. The associations with smoking status and dietary adherence further suggest that comprehensive obesity management should incorporate behavioural risk assessment and individualized lifestyle support alongside pharmacological therapy. Overall, these findings support continued monitoring of medication‐use patterns, reinforcement of evidence‐based prescribing practices, and development of targeted public health strategies to promote safe, informed and sustainable use of antidiabetic medications for weight management.

The findings of this study have several implications for clinical practice and public health policy. The observed rates of unsupervised medication use, reported adverse effects and weight regain following treatment discontinuation highlight the need for physician‐guided prescribing, pharmacist‐led counselling and strengthened pharmacy oversight to discourage inappropriate use. Community pharmacists are particularly well positioned to identify inappropriate over‐the‐counter access, reinforce appropriate prescribing practices, educate patients regarding expected adverse effects and encourage timely referral to physicians when necessary [17]. Public awareness campaigns focusing on the benefits, risks, expected adverse effects and long‐term nature of obesity management may improve informed decision‐making among potential users. Furthermore, strengthening prescription‐monitoring systems, enhancing regulatory oversight of medication dispensing, and developing multidisciplinary obesity management programs involving physicians, pharmacists, dietitians and behavioural specialists may improve both the safety and effectiveness of pharmacological weight management in Lebanon.

Although social media has emerged as a key source of information regarding GLP‐1 receptor agonists and public perceptions of their use [11, 12], the present study did not directly assess participants' primary information sources, frequency of exposure or trust in online content. Consequently, the influence of digital media on participants' knowledge, attitudes and medication‐use behaviours could not be evaluated and should be investigated in future studies. Collectively, these findings provide important baseline evidence to support evidence‐based prescribing practices, patient education and future policy initiatives aimed at promoting the safe and appropriate use of antidiabetic medications for weight management in Lebanon.

### 4.1 Strengths and Limitations

This study has several notable strengths. It is among the first to comprehensively assess KAP regarding the use of antidiabetic medications for weight loss in Lebanon, providing important region‐specific insights. The study included a relatively large and diverse sample representing different governorates, urban and rural regions, and varied socioeconomic and educational backgrounds, allowing exploration of medication‐use patterns across diverse participant characteristics. The questionnaire captured a broad range of factors, including lifestyle behaviours, clinical history and other behavioural characteristics, allowing for a multidimensional assessment of variables associated with medication use. Additionally, the inclusion of participants from both medical and non‐medical sectors enabled meaningful comparisons across different levels of health literacy.

Several limitations should be considered when interpreting the findings of this study. First, the cross‐sectional design precludes any inference of causality between knowledge, attitudes and practices related to the use of antidiabetic medications for weight loss; therefore, only associations can be reported. Consequently, the observed relationships should not be interpreted as directional effects. For example, higher knowledge scores among participants who used antidiabetic medications may reflect greater exposure to medication‐related information among medication users rather than knowledge leading to medication use. Similarly, associations involving smoking status, dieting behaviour and prior weight‐control complications may reflect reverse causation or residual confounding from unmeasured factors. Longitudinal studies are needed to clarify the temporal direction and causal nature of these relationships.

Second, the study relied on self‐reported data, which may be subject to recall bias and social desirability bias. Participants may have overestimated their knowledge levels or underreported unsafe or non‐medically supervised medication use, potentially affecting the accuracy of the findings. In addition, the absence of objective verification of body mass index and medication use through clinical records may have introduced potential misclassification. Although unsupervised medication use and treatment‐related adverse effects were valuable findings, subgroup analyses comparing supervised and unsupervised users with respect to side effects, weight‐loss outcomes or weight regain were not performed. The relatively small number of medication users limited statistical power and reduced the reliability of such comparisons. In addition, although more detailed descriptive characterization of medication users (e.g., according to age, sex, body mass index, obesity status, diabetes status and medication type) would have provided valuable context for interpreting medication‐use patterns, the relatively small number of users limited the feasibility and interpretability of such subgroup analyses. Future studies with larger samples of medication users should further characterize this population.

Third, the online convenience sampling strategy may have introduced selection bias (Bethlehem [18]), with likely overrepresentation of younger individuals, those with higher educational attainment, and participants employed in medical‐related fields. The study population was characterized by a particularly high proportion of respondents with university or postgraduate education (87.4%) and employment in medical‐related professions (57.0%), which exceeds what would generally be expected in the Lebanese adult population. Moreover, our multivariable analyses demonstrated that both higher educational attainment and employment in medical‐related fields were independently associated with greater knowledge and more appropriate attitudes toward the safe use of antidiabetic medications for weight loss. Therefore, these characteristics may have contributed to higher observed knowledge and attitude scores within the study sample. Individuals with greater internet access and stronger interest in health‐related topics may have been more likely to participate, while those with limited digital literacy or restricted internet access may have been underrepresented. As a result, the sample may not be fully representative of the Lebanese adult population, and the findings may overestimate levels of knowledge and awareness regarding antidiabetic medications used for weight loss. In addition, non‐response bias cannot be excluded because information regarding individuals who chose not to participate was unavailable, preventing comparison between respondents and non‐respondents.

Fourth, although several relevant variables were included in the analysis, residual confounding from unmeasured factors cannot be excluded. Important factors potentially associated with the use of antidiabetic medications for weight loss, including obesity severity, body‐image concerns, psychological distress, motivation toward weight management, healthcare accessibility, health literacy, cultural beliefs, weight stigma and exposure to social media, were not assessed in this study. These factors may be associated with participants' knowledge, attitudes and medication‐use behaviours and may partially explain some of the observed associations. Furthermore, exposure to social media content specifically related to antidiabetic medications for weight loss was not directly measured, despite its potential relevance in shaping medication awareness, perceptions and use patterns.

Fifth, the exploratory nature of the bivariate analyses without formal adjustment for multiple comparisons may have increased the risk of Type I error; therefore, some statistically significant associations should be interpreted with caution. In addition, variable selection for the multivariable regression models relied primarily on exploratory bivariate screening. Although this approach was used to identify candidate variables associated with the study outcomes, it may not have captured all theoretically relevant confounders. Clinically relevant variables such as age, sex, body mass index, obesity status and chronic disease status, which are well‐established determinants of obesity pharmacotherapy, may not have been retained in the final models despite their potential confounding effects. Consequently, residual confounding and model instability cannot be excluded. Future studies should consider theory‐driven model‐building strategies that incorporate clinically relevant variables irrespective of their statistical significance. Furthermore, formal assessment and reporting of regression model assumptions and performance were not undertaken. Specifically, multicollinearity diagnostics, residual analyses, assessment of normality and homoscedasticity (for linear regression models), goodness‐of‐fit measures and model performance statistics were not systematically evaluated. Consequently, although the regression models identified statistically significant associations, the robustness and predictive performance of these models cannot be fully established, which limits the interpretation of these findings.

Finally, although the questionnaire was adapted from previously published instruments, reviewed for content validity, and pilot‐tested before implementation, formal psychometric validation was not performed. Specifically, internal consistency measures (e.g., Cronbach's alpha), construct validity assessment, and exploratory or confirmatory factor analyses were not conducted. Consequently, the reliability and validity of the knowledge and attitude scales cannot be fully established, which may affect the precision of the reported associations. Additionally, the categorization of knowledge and attitude levels was based on Bloom's cut‐off criteria, which is commonly used in KAP studies; however, no validated scoring system specific to antidiabetic medications used for weight loss currently exists. Additionally, because formal psychometric validation was not performed, the reliability and construct validity of the knowledge and attitude scales could not be fully established. The multivariable analyses were exploratory and based primarily on variables identified through bivariate analyses rather than theory‐driven model building, which may have resulted in residual confounding. Furthermore, information regarding participants' primary sources of medication‐related information, including social media exposure and health‐information‐seeking behaviours, was not collected and therefore could not be evaluated.

Future studies should consider longitudinal and prospective designs to better establish temporal relationships between knowledge, attitudes and medication‐use behaviours. In addition, studies using probability‐based sampling methods, validated questionnaires with formal psychometric evaluation, objective verification of medication use and anthropometric measurements, and direct assessment of social media exposure would provide stronger evidence and improve the generalizability of findings to the Lebanese adult population.

### 4.2 Study Perspectives

Based on the findings of the present study, several perspectives and recommendations can be considered:

1. Public health campaigns: Healthcare stakeholders should implement evidence‐based campaigns to increase public knowledge regarding antidiabetic medications, highlighting both their benefits and potential risks in the context of weight management.
2. Surveillance and misinformation control: Authorities should establish strict monitoring to address misconceptions and prevent the dissemination of unverified information, ensuring that the population receives guidance grounded in medical evidence rather than personal experiences.
3. Promotion of healthy lifestyles: Healthcare professionals should emphasize the importance of diet, physical activity and overall lifestyle modifications to prevent obesity and associated health complications.
4. Dietitian consultation: The public should be encouraged to seek guidance from dietitians for personalized planning, implementation and evaluation of healthy lifestyle strategies, as well as for safe and supervised use of antidiabetic medications for weight management.
5. Regulatory enforcement: Policies should restrict over‐the‐counter sales of antidiabetic medications, ensuring that these medications are dispensed only with valid medical prescriptions.
6. Targeted interventions: Healthcare organizations should develop programs for high‐risk populations, including smokers, individuals with sedentary occupations, and those with lower health literacy to improve knowledge and support informed decision‐making.
7. Future research on long‐term effects: Further studies should evaluate the sustainability, safety and potential for weight regain following cessation of antidiabetic medications. Future longitudinal studies should incorporate validated questionnaires with formal psychometric evaluation, theory‐driven analytical models, objective verification of medication use, detailed assessment of information sources including social media exposure and larger samples of medication users to better characterize determinants of medication utilization and long‐term outcomes.
8. Qualitative investigations: Researchers should conduct qualitative studies to explore the psychological, behavioural and perceptual factors underlying individuals' attitudes and practices regarding the use of antidiabetic medications for weight loss.

## 5 Conclusion

This study identified several behavioural and clinical factors associated with the use of antidiabetic medications for weight loss among Lebanese adults, including smoking status, previous weight‐related complications, higher knowledge levels and adherence to dietary strategies. These findings suggest that the use of pharmacological weight management strategies is more common among individuals actively engaged in weight‐control behaviours or those who have experienced difficulties achieving weight loss through conventional approaches.

Importantly, higher knowledge was associated with medication use, which may reflect greater awareness and familiarity with these therapies, increased exposure through healthcare or media sources, or prior personal experience with their use. Similarly, smoking status and dietary behaviours were associated with medication use; however, owing to the cross‐sectional design, causal or temporal relationships cannot be established. In addition, individuals with sedentary occupational roles were less likely to use these medications, although the underlying reasons for this association remain unclear and warrant further investigation.

Obesity represents a major and growing global public health challenge, with increasing use of antidiabetic medications for weight management, including among non‐diabetic individuals. This study is the first in Lebanon to comprehensively assess knowledge, attitudes and practices regarding these medications, providing important insights into public awareness, perceptions and behaviours in this context. The findings highlight potential risks associated with inappropriate or unsupervised use, including gastrointestinal adverse effects and more serious complications such as kidney disease and thyroid C‐cell tumours. These observations underscore the importance of medical supervision, appropriate prescribing and safe medication use.

Overall, this study contributes to a better understanding of the KPA and factors associated with the use of antidiabetic medications for weight management in Lebanese adults The results can inform targeted educational interventions, support healthcare providers in improving patient counselling, and guide public health policies aimed at promoting safe and evidence‐based use of these medications. Furthermore, the findings emphasize the need for awareness campaigns addressing misconceptions and promoting proper medical follow‐up. Future longitudinal research is needed to clarify temporal relationships and better understand the determinants of medication used for weight management.

## Author Contributions

**Rim Masri:** conceptualization, data curation, supervision, project administration, writing – original draft, writing – review and editing, investigation, formal analysis. **Pascale Salameh:** data curation, investigation, formal analysis, writing – review and editing, supervision. **Hekmat Kaakour:** data curation, investigation, writing – original draft, formal analysis. **Hasnaa Hamdan:** data curation, investigation, formal analysis, writing – original draft.

## Funding

The authors have nothing to report.

## Ethics Statement

This study was approved by the Institutional Review Board of the Lebanese Hospital Geitaoui–University Medical Center (IRB Approval Number: 2025‐IRB‐012; Approval Date: 19 March 2025). The study was conducted in accordance with the ethical principles outlined in the Declaration of Helsinki.

## Consent

All participants were informed about the objectives and procedures of the study, and electronic informed consent was obtained prior to participation. Confidentiality and anonymity were strictly maintained, and participation was voluntary, with the right to withdraw at any time without consequences.

## Conflicts of Interest

The authors declare no conflicts of interest.

## Supporting information


**Table S1:** Knowledge about antidiabetic medications for weight loss among the study population.
**Table S2:** Knowledge score about antidiabetic medications for weight loss among the study population.
**Table S3:** Attitude score toward the use of antidiabetic medications for weight loss among the study population.

## Data Availability

The data that support the findings of this study are available on request from the corresponding author. The data are not publicly available due to privacy or ethical restrictions.
