# Supplementary material for: Knowledge, Attitudes and Practices on the Use of Antidiabetic Medications for Weight Loss: A Cross‐Sectional Study in the Lebanese Population
Source: Endocrinol Diabetes Metab. 2026 Jul 14;9(4):e70286. doi: 10.1002/edm2.70286 (PMC13367752; doi:10.1002/edm2.70286)
Supplement: Supplementary file 1 — Table S1: Knowledge about antidiabetic medications for weight loss among the study population. Table S2: Knowledge score about antidiabetic medications for weight loss among the study population. Table S3: Attitude score toward the use of antidiabetic medications for weight loss among the study population. [file EDM2-9-e70286-s001.docx]

**Supplementary material**

Table S1. Knowledge about antidiabetic medications for weight loss among the study population.

|  | | Frequency | Percent |
| --- | --- | --- | --- |
| To the best of your knowledge, which of the following medications can be used to lose weight? | Ozempic® * | 277 | 68.4 |
|  | Saxenda® * | 131 | 32.3 |
|  | Mounjaro® * | 116 | 28.6 |
|  | Glucophage® * | 212 | 52.3 |
|  | Insulin | 33 | 8.1 |
|  | Amaryl® | 12 | 3.0 |
|  | Jardiance® | 25 | 6.2 |
| Which of the following medications is approved to be used for weight loss by regulatory authorities such as the Food and Drug Administration? | Ozempic® * | 239 | 59.0 |
|  | Saxenda® * | 96 | 23.7 |
|  | Mounjaro® | 89 | 22.0 |
|  | Glucophage® | 137 | 33.8 |
|  | Insulin | 26 | 6.4 |
|  | Amaryl® | 6 | 1.5 |
|  | Jardiance® | 7 | 1.7 |
| To the best of your knowledge, what are the side effects of the injectable medications used for weight loss? | Nausea and vomiting * | 297 | 73.3 |
|  | Diarrhea * | 196 | 48.4 |
|  | Abdominal pain * | 193 | 47.7 |
|  | Headache | 158 | 39.0 |
|  | Fatigue | 188 | 46.4 |
|  | Weight gain | 21 | 5.2 |
|  | Skin swelling or irritation where the needle was inserted | 111 | 27.4 |
|  | Depression | 147 | 36.3 |
|  | Pancreatitis | 123 | 30.4 |
|  | Tumors such as thyroid tumors | 74 | 18.3 |
|  | Urinary tract infection | 39 | 9.6 |
| Among the following items, which are strategies that can be used to lose weight? | Diet * | 374 | 92.3 |
|  | Exercise * | 365 | 90.1 |
|  | Herbal products | 71 | 17.5 |
|  | Pharmaceutical drugs * | 129 | 31.9 |
|  | Cognitive and behavioral therapy | 113 | 27.9 |
|  | Surgery * | 177 | 43.7 |

* Correct answers

Amaryl® (glimepiride), Jardiance® (empagliflozin), Ozempic® (semaglutide), Saxenda® (liraglutide), Mounjaro® (tirzepatide), Glucophage® (metformin).

Table S2. Knowledge score about antidiabetic medications for weight loss among the study population.

|  | | Knowledge |
| --- | --- | --- |
| N | | 405 |
| Mean | | 69.39 |
| Median | | 70.97 |
| Std. Deviation | | 8.71 |
| Minimum | | 45.16 |
| Maximum | | 93.55 |
| Percentiles | 25 | 64.52 |
|  | 50 | 70.97 |
|  | 75 | 74.19 |

Table S3. Attitude score towards the use of antidiabetic medications for weight loss among the study population.

|  | | Attitude |
| --- | --- | --- |
| N | | 405 |
| Mean | | 69.11 |
| Median | | 69.33 |
| Std. Deviation | | 5.54 |
| Minimum | | 54.67 |
| Maximum | | 89.33 |
| Percentiles | 25 | 65.33 |
|  | 50 | 69.33 |
|  | 75 | 72.00 |
